# Symptom networks in glioma patients: understanding the multidimensionality of symptoms and quality of life

**DOI:** 10.1007/s11764-023-01355-8

**Published:** 2023-03-16

**Authors:** J. G. Röttgering, T. M. C. K. Varkevisser, M. Gorter, V. Belgers, P. C. De Witt Hamer, J. C. Reijneveld, M. Klein, T. F. Blanken, L. Douw

**Affiliations:** 1 Cancer Center Amsterdam, Brain Tumor Center, Amsterdam, The Netherlands; 2 Amsterdam UMC Location Vrije Universiteit Amsterdam, Medical Psychology, Boelelaan 1117, Amsterdam, The Netherlands; 3 Amsterdam UMC Location Vrije Universiteit Amsterdam, Anatomy and Neurosciences, Boelelaan 1117, Amsterdam, The Netherlands; 4 Amsterdam UMC Location Vrije Universiteit Amsterdam, Neurology, Boelelaan 1117, Amsterdam, The Netherlands; 5 Amsterdam UMC Location Vrije Universiteit Amsterdam, Neurosurgery, Boelelaan 1117, Amsterdam, The Netherlands; 6 Department of Neurology, SEIN, Heemstede, The Netherlands; 7 Department of Psychological Methods, University of Amsterdam, 1018 WT Amsterdam, The Netherlands

**Keywords:** Fatigue, Quality of life, Mental health, Brain neoplasms, Patient-reported outcome measures, Network analysis

## Abstract

**Purpose:**

To comprehend the complex relationship between symptoms and health-related quality of life (HRQoL) in patients with diffuse glioma, we applied symptom network analysis to identify patterns of associations between depression, cognition, brain tumor-related symptoms, and HRQoL. Additionally, we aimed to compare global strength between symptom networks to understand if symptoms are more tightly connected in different subgroups of patients.

**Methods:**

We included 256 patients and stratified the sample based on disease status (preoperative vs. postoperative), tumor grade (grade II vs. III/IV), and fatigue status (non-fatigued vs. fatigued). For each subgroup of patients, we constructed a symptom network. In these six networks, each node represented a validated subscale of a questionnaire and an edge represented a partial correlation between two nodes. We statistically compared global strength between networks.

**Results:**

Across the six networks, nodes were highly correlated: fatigue severity, depression, and social functioning in particular. We found no differences in GS between the networks based on disease characteristics. However, global strength was lower in the non-fatigued network compared to the fatigued network (5.51 vs. 7.49, *p* < 0.001).

**Conclusions:**

Symptoms and HRQoL are highly interrelated in patients with glioma. Interestingly, nodes in the network of fatigued patients were more tightly connected compared to non-fatigued patients.

**Implications for Cancer Survivors:**

We introduce symptom networks as a method to understand the multidimensionality of symptoms in glioma. We find a clear association between multiple symptoms and HRQoL, which underlines the need for integrative symptom management targeting fatigue in particular.

**Supplementary Information:**

The online version contains supplementary material available at 10.1007/s11764-023-01355-8.

## Introduction

Having a brain tumor significantly impacts well-being, though how symptoms emerge, impact each other, and are related to health-related quality of life (HRQoL) is still poorly understood [1]. Glioma patients often suffer from more than ten symptoms simultaneously, including fatigue, depression, and cognitive deficits, and these symptoms might also influence one another [1, 2]. As an illustration: glioma patients often suffer from cognitive deficits related to the tumor and therapy, which could result in a higher mental load when completing tasks, leading to fatigue, and in turn potentially resulting in social isolation. Research has provided many insights into which sociodemographic factors, disease characteristics, and neurocognitive and psychological symptoms are correlated with, or can predict, any individual symptom. However, shifting our focus from single symptoms to the interaction between multiple different symptoms and HRQoL could improve our understanding of the complexity of symptoms in glioma.

In line with this paradigm shift, symptom networks are a rather novel application of network analysis used to quantify how multiple symptoms co-occur and are associated with each other [3–5]. In general, a network is defined as a collection of nodes that are connected by edges, together forming a graph. In a symptom network, nodes represent subscales or items or sum scores of patient-reported outcome measures (PROMs), while edges represent the associations between these variables on a group level [3, 4]. In recent years, symptom networks have been applied to investigate symptoms in patients with different cancer types [6, 7]. For example, cancer symptom networks of patients on chemotherapy revealed that symptoms are indeed highly intercorrelated and that nausea, loss of appetite, and diminished energy play an important role in these networks [8].

In addition to the research that has been done in non-CNS cancer patients, investigating symptom networks in brain tumor patients seems particularly interesting, as the tumor itself also exerts a direct effect on brain function, inducing or exacerbating specific neurological and cognitive symptoms [1, 9]. To comprehend how symptomatology differs throughout the course of the disease or varies between tumor types, symptom networks of different groups of patients can also be compared to each other [7, 10]. A study applying this approach found that fatigue and cognition are closely related in breast cancer symptom networks, but not in leukemia symptom networks [7]. Results like these might lead to the development of interventions for specific patient groups, for example focusing on treating fatigue and cognition simultaneously. Glioma is a progressive and infiltrative type of brain cancer with a consequently high symptom burden. Clinically, the disease trajectory is marked by different phases, including treatment and active follow-up. There are large differences in survival between patients with different tumor grades [11]. Comparing symptom networks of glioma patients with different disease characteristics would be of interest to understand when symptom management should be initiated, for which symptoms, and for whom.

In the context of glioma symptom networks, fatigue is a specifically interesting symptom to explore, because it is the most prevalent and burdensome symptom, and is highly correlated to other symptoms such as depression and subjective cognitive complaints [1, 12]. Patients with cancer also often suffer from fatigue, and the node plays an important role within cancer symptom networks: it is highly connected to symptoms like distress, pain, and drowsiness [7, 10, 13, 14]. It has been suggested that targeting or treating these highly connected symptoms might result in the improvement of other symptoms, although this remains to be proven with intervention studies [15]. Clinically, understanding the role of fatigue in relation to other symptoms and elucidating whether symptoms of fatigued and non-fatigued patients are intercorrelated differently could be of benefit for glioma symptom management.

Therefore, we applied symptom network analysis to study the interrelatedness of questionnaires on fatigue, depression, subjective cognitive complaints, several brain tumor-related symptoms, and HRQoL. Additionally, we aimed to compare symptom networks of subgroups of patients based on disease status, tumor grade, and fatigue status.

## Methods

### Patients and setting

We have retrospectively combined questionnaire data from several observational studies performed between 2009 and 2021 at Amsterdam UMC location Vrije Universiteit Amsterdam, a tertiary referral hospital for neuro-oncological care in the Netherlands. Ten manuscripts have been published on this data, mainly focusing on imaging, neurophysiology, and cognition (Supplementary Table S1, [16–25]). Ethical approval for the studies was granted by the Medical Ethics Review Committee of Amsterdam UMC location Vrije Universiteit Amsterdam (METc VUmc 2008.52; 2009.189; 2010.126; 2014.297), and written informed consent had been obtained from the patients. One study was a retrospective observational study, aggregating data that was collected as part of standard clinical care [25], and the other studies were prospective observational studies. For the current study, we included adult patients with histopathologically confirmed WHO grade II, III, or IV glioma who had completed a set of PROMs on fatigue, depression, subjective cognitive complaints, brain-tumor-related symptoms, and HRQoL at least once [26]. All included patients were able and willing to participate in research and visit the hospital, which resulted in a sample of patients with predominantly lower-grade tumors and relatively high functional performance status.

### Patient-reported outcome measures

*Fatigue* was assessed with the Checklist Individual Strength (CIS), consisting of the validated subscales fatigue severity, concentration problems, reduced motivation, and reduced activity level [27]. A cut score  ≥ 27 on the fatigue severity subscale was applied to define an elevated level of fatigue [27]. *Depression* was assessed with the sum score of the Center for Epidemiologic Studies Depression questionnaire (CES-D) and *self-perceived cognitive functioning* with the sum score of the Medical Outcomes Study Cognitive Functioning Scale (MOS-Cog  [28, 29]). *Brain tumor–related symptoms* were assessed with the European Organization for Research and Treatment of Cancer brain tumor module (BN-20) [30]. This questionnaire consists of several validated subscales, of which we have included the subscales future uncertainty, visual disorder, motor dysfunction, communication deficits, headaches, seizures, and drowsiness. *HRQoL* was assessed with the 36-Item Short Form Survey (SF-36). This questionnaire consists of several validated subscales including physical functioning, social functioning, role limitations due to physical health problems, role limitations due to emotional problems, emotional well-being, bodily pain, general health perceptions, changes in health, and energy/fatigue [31]. We did not include the energy/fatigue item to avoid redundancy with the CIS. All questionnaires were scored according to the relevant scoring manuals. The subscales and sum scores of these questionnaires were used to compute symptom networks with 21 nodes. Each node reflected a validated subscale or sum score of a questionnaire (see Supplementary Table S2).

### Computing symptom networks

**Fig. 1:**
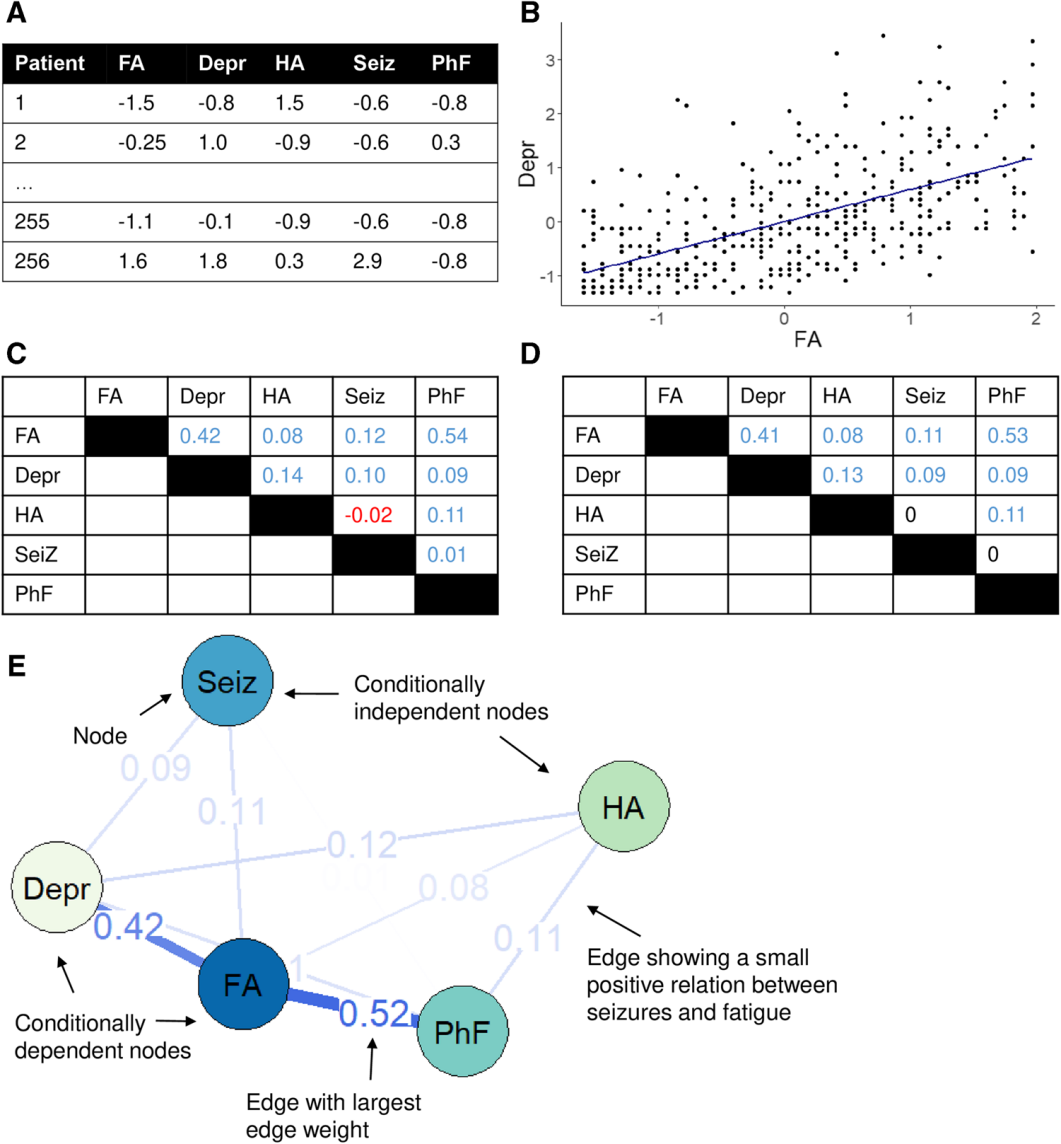
Example of the construction of a symptom network. **A** *Z*-scores of five symptoms, data per patient. **B** Scatterplot between fatigue and depression. **C** Group-level Spearman’s partial correlation matrix. **D** Partial correlation matrix after regularization. **E** Symptom network. For example, the strength of the node Seizures is 0.11 + 0.09 = 0.20. Abbreviations: FA, fatigue; Depr, depression; HA, headaches; Seiz, seizures; PhF, physical functioning

To compute these symptom networks, we took several consecutive steps, which is exemplified in Fig. 1. First, we scaled the questionnaire data for comparability (Fig. 1A). Then, we constructed a group-level Spearman’s partial correlation matrix (Fig. 1B and C). As is customary when estimating symptom networks with a relatively large number of nodes, we applied a regularization technique to the matrix to exclude possible spurious edges (Fig. 1D [32]). Finally, we visualized the regularized matrix as a symptom network (Fig. 1E). The resulting network can be inspected visually, and in the example we can see that fatigue and depression are strongly connected [33]. To further interpret how strongly nodes are connected to other nodes, node strength can be calculated by taking the sum of the edges connecting a particular node to other nodes (Fig. 1E). Additionally, since a large number of parameters are estimated, accuracy of the edge weights and stability of node strength should be assessed (see Supplementary materials for a more detailed explanation, [34]).

### Subgroup analyses

The available dataset consisted of 256 patients, who had completed 420 assessments at different time points. Since we aimed to compare symptom networks based on disease status, tumor grade, and fatigue status for each of the three sub-questions, the entire sample of 420 assessments was divided into two subgroups a total of three times. First, all assessments were split in *preoperative assessments* (subgroup 1A) and *postoperative assessments* (subgroup 1B). Note that assessments in patients with progressive disease were excluded from subgroup 1B, so this subgroup consisted only of patients on active treatment or with stable disease and under monitoring for tumor growth. Second, to assess symptom networks between tumor grades, we split the entire sample in *patients with a grade II tumor* (subgroup 2A), and *patients with a grade III or IV tumor* (subgroup 2B). Finally, we split the entire sample based on the CIS-fatigue cut-off score, resulting in *non-fatigued patients* (subgroup 3A), and *fatigued patients* (subgroup 3B). For each of the subgroups, only one assessment per patient was included to avoid within-subject duplicates, so only one assessment per patient was included in each network. See Supplementary Figure S1 for additional information on how we selected the subgroups.

### Statistical analysis

Analyses were conducted using Rstudio (version 4.0.3 [35]). The scripts for the statistical analyses are available in an online repository (https://github.com/multinetlab-amsterdam/projects/tree/master/symptomnetwork_glioma_paper_2022) and can be run with the provided synthetic data [36].

For each of the six subgroups, we computed a symptom network with Gaussian graphical models based on Spearman’s partial correlation matrices. Networks were regularized with EBICglasso with a tuning parameter of 0.25. Here, we chose a lower value of the tuning parameter, resulting in a higher number of estimated edges, thereby possibly including some spurious edges [4, 37]. The disease status (1A, 1B) and tumor grade (2A, 2B) networks consisted of 21 nodes. The fatigue status (3A, 3B) networks consisted of only 17 nodes because the CIS nodes were excluded as the sample was already stratified based on the CIS-fatigue score [38]. For each of the networks, strength per node was calculated, as well as the accuracy of the edge weights and the stability of nodal strength (see Supplementary Methods for a detailed explanation [4, 34]).

To understand whether network density was different between subgroup networks (1A versus 1B, 2A versus 2B, and 3A versus 3B), we compared global strength (GS) using permutation-based network comparison tests [39]. Global strength is the average node strength across a network and can be used as a measure of how tightly symptoms are interconnected [39]. Furthermore, similarity between two networks was quantified using Spearman’s correlations between edge weights of the networks [14].

Additionally, we performed two sensitivity analyses for comparing global strength between subgroups: excluding the CIS nodes from networks 1A and 1B and excluding assessments from patients that contributed more than one assessment. Since the fatigue networks consisted of 17 nodes and the other networks of 21 nodes, we excluded the CIS nodes from the 1A preoperative and 1B postoperative networks and compared GS between the two. As described in the “Subgroup analyses” section above, if a patient had conducted two assessments, for example, one preoperative and one postoperative, the corresponding assessment was included in both subgroups 1A and 1B. To ensure including multiple assessments did not influence the permutation test to compare global strength, we removed patients with multiple assessments so each patient contributed only one data point per comparison, so to subgroups 1A or 1B, to subgroup 2A or 2B, and to 3A or 3B. After the exclusion of these assessments, global strength was compared again between networks.

## Results

Of the 256 included patients, the mean age was 47 years, with 63% males, and 47% of the patients had a grade II tumor, and 25% and 28% had a grade III or IV tumor, respectively. The size of the six subgroups ranged between 117 and 174 patients (Table 1), and the outcomes of the questionnaires of the subgroups are presented in Supplementary Table S3.

**Table 1:** Sociodemographic and clinical characteristics

	1A. Preoperative (*N* = 166)	1B. Postoperative (*N* = 146)	2A. Tumor grade II (*N* = 120)	2B. Tumor grades III and IV (*N* = 136)	3A. Non-fatigued (*N* = 117)	3B. Fatigued (*N* = 174)
Age, mean (SD)	45.9 (14.1)	44.7 (12.1)	41.5 (11.7)	50.9 (13.4)	45.1 (13.6)	46.8 (13.2)
Sex, n (%)						
Male	103 (62.0%)	89 (61.0%)	68 (56.7%)	92 (67.6%)	83 (70.9%)	99 (56.9%)
Female	63 (38.0%)	57 (39.0%)	52 (43.3%)	44 (32.4%)	34 (29.1%)	75 (43.1%)
Tumor hemisphere, n (%)						
Left	101 (60.8%)	82 (56.2%)	69 (57.5%)	77 (56.6%)	76 (65.0%)	89 (51.1%)
Right	61 (36.7%)	60 (41.1%)	47 (39.2%)	54 (39.7%)	39 (33.3%)	77 (44.3%)
Both	4 (2.4%)	4 (2.7%)	4 (3.3%)	5 (3.7%)	2 (1.7%)	8 (4.6%)
Tumor grade and histology, n (%)						
II astrocytoma	42 (25.3%)	45 (30.8%)	65 (54.2%)	0 (0%)	36 (30.8%)	42 (24.1%)
II oligoastrocytoma*	13 (7.8%)	11 (7.5%)	16 (13.3%)	0 (0%)	5 (4.3%)	12 (6.9%)
II oligodendroglioma	24 (14.5%)	29 (19.9%)	39 (32.5%)	0 (0%)	18 (15.4%)	27 (15.5%)
III astrocytoma	13 (7.8%)	10 (6.8%)	0 (0%)	21 (15.4%)	9 (7.7%)	16 (9.2%)
III oligoastrocytoma*	4 (2.4%)	3 (2.1%)	0 (0%)	6 (4.4%)	4 (3.4%)	3 (1.7%)
III oligodendroglioma	24 (14.5%)	21 (14.4%)	0 (0%)	36 (26.5%)	14 (12.0%)	27 (15.5%)
IV glioblastoma	46 (27.7%)	27 (18.5%)	0 (0%)	73 (53.7%)	31 (26.5%)	47 (27.0%)
IDH status, n (%)						
IDH wildtype	36 (21.7%)	12 (8.2%)	9 (7.5%)	38 (27.9%)	18 (15.4%)	31 (17.8%)
IDH mutant	72 (43.4%)	71 (48.6%)	77 (64.2%)	31 (22.8%)	56 (47.9%)	72 (41.4%)
Unknown	58 (34.9%)	63 (43.2%)	34 (28.3%)	67 (49.3%)	43 (36.8%)	71 (40.8%)
Disease status, n (%)						
Preoperative	166 (100%)	0 (0%)	62 (51.7%)	76 (55.9%)	56 (47.9%)	91 (52.3%)
On active treatment	0 (0%)	68 (46.6%)	24 (20.0%)	29 (21.3%)	25 (21.4%)	39 (22.4%)
Stable^	0 (0%)	78 (53.4%)	30 (25.0%)	20 (14.7%)	29 (24.8%)	34 (19.5%)
Progression	0 (0%)	0 (0%)	4 (3.3%)	11 (8.1%)	7 (6.0%)	10 (5.7%)

^*^Histological diagnosis of these patients was based on the older 2007 WHO classification of central nervous system tumors [40]

^Stable disease was defined as no radiological or clinical progression and no anti-tumor therapy at least 3 months prior to the assessment

### Symptom network in preoperative patients

**Fig. 2:**
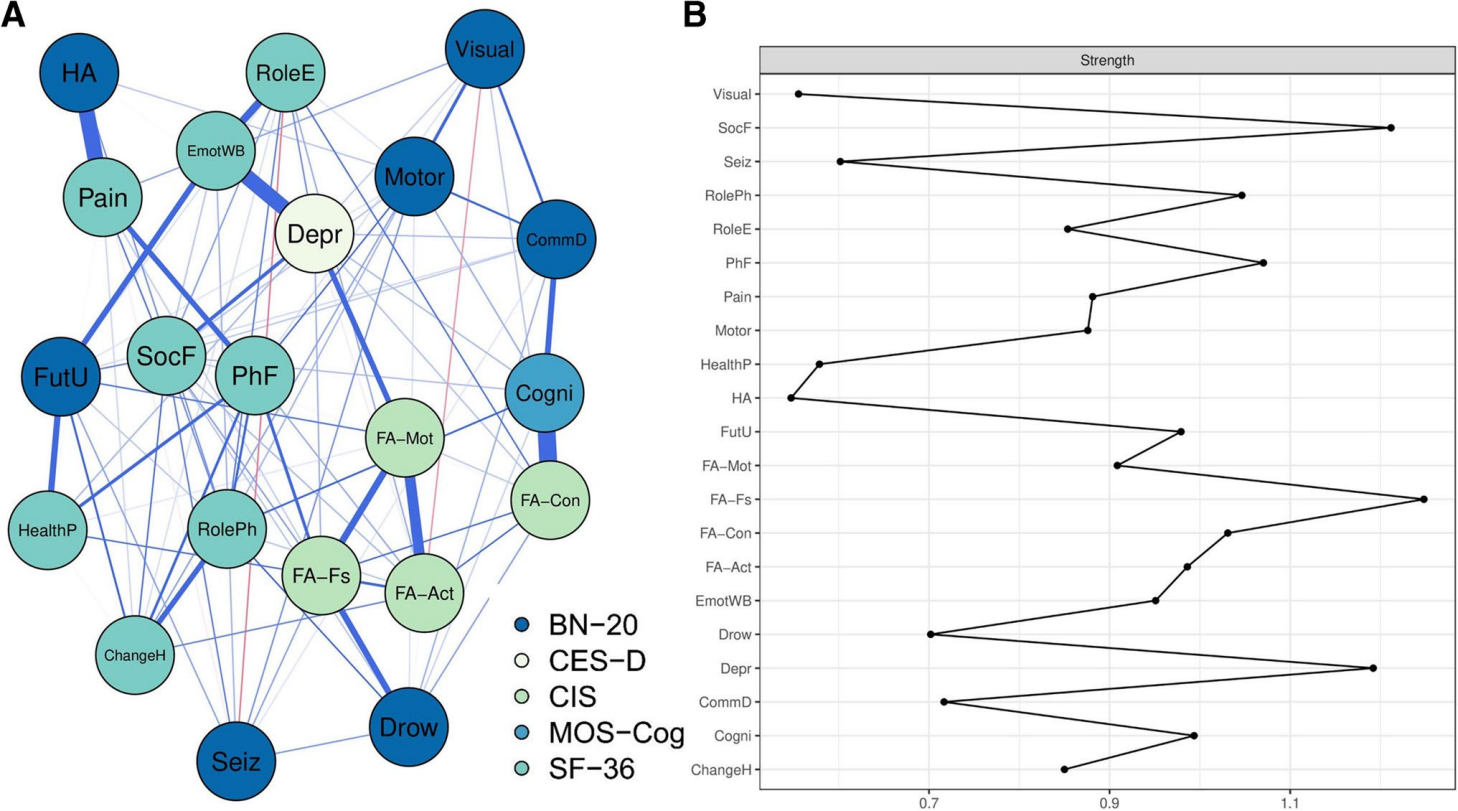
Symptom network of the preoperative patients (subgroup 1A). **A** Symptom network. The colors of the nodes refer to the corresponding questionnaire. A blue line indicates a positive relationship between two nodes and a red line a negative relationship. Line width is proportional to edge weight. **B** Node strength. For example, the HA node has a relatively low strength of 0.088 + 0.40 + 0.044 = 0.53. Abbreviations: BN-20, European Organization for Research and Treatment of Cancer brain tumor module; ChangeH, SF-36 Change in health; CIS, Checklist Individual Strength; Cogni, Medical Outcomes Study Cognitive Functioning Scale (MOS-Cog); CommD, BN-20 communication deficit; Depr, Center for Epidemiologic Studies Depression questionnaire (CES-D); Drow, BN-20 drowsiness; EmotWB, SF-36 Emotional well-being; FA-Act, CIS reduced activity level; FA-con, CIS concentration problems; FA-Fs, CIS fatigue severity; FA-Mot, CIS reduced motivation; FutU, BN-20 future uncertainty; HA, BN-20 headaches; HealthP, SF-36 General health perception; Motor, BN-20 motor dysfunction; Pain, SF-36 Pain; PhF, SF-36 Physical functioning; RoleE, SF-36 Role limitations due to emotional problems; RolePh, SF-36 Role limitations due to physical health; Seiz, BN-20 seizures; SF-36, 36-Item Short Form Survey; SocF, SF-36 Social functioning; Visual, BN-20 visual disorder

First, we present the symptom network of the **preoperative patients** (subgroup 1A, see Fig. 2A) to guide the interpretation of these glioma symptom networks. This network consisted of 98 edges connecting the 21 nodes. The nodes *CIS-fatigue*, *SF-36 social functioning*, and *CES-D depressive symptoms* were strongly connected to other symptoms (node strength = 1.25, 1.21, 1.19, respectively, Fig. 2B). Stability checks also implicated that node strength could be interpreted accurately (see Supplementary Figure S2.B). Strong edges were present between *subjective cognitive complaints* and *CIS-concentration*, *SF-36 bodily pain* and *BN-20 headaches*, and *CES-D depressive symptoms* and *SF-36 emotional well-being* (edge weights 0.44, 0.40, 0.39 respectively); however, edge weights should be interpreted with some caution because of large confidence intervals (see Supplementary Table S4 and Supplementary Figure S2.C).

### Comparing symptom networks based on disease status and tumor grade

The symptom networks of the subgroups based on disease status and tumor grade are presented in Fig. 31A, 2B. Across these networks, *fatigue severity*, *subjective cognitive complaints*, *depressive symptoms*, *social functioning*, and *physical functioning* were highly connected to other symptoms (Supplementary Figures S2-S7). Global strength was not statistically different between the preoperative (subgroup 1A) and postoperative (subgroup 1B) networks (GS = 9.34 vs. GS = 9.21, *p* = 0.67), as well as between the networks based on tumor grade (*p* = 0.28). Furthermore, the edge weights of disease status subgroup networks were moderately correlated (*ρ* = 0.54, *p* < 0.001), similar to the tumor grade networks (*ρ* = 0.51, *p* < 0.001).

**Fig. 3:**
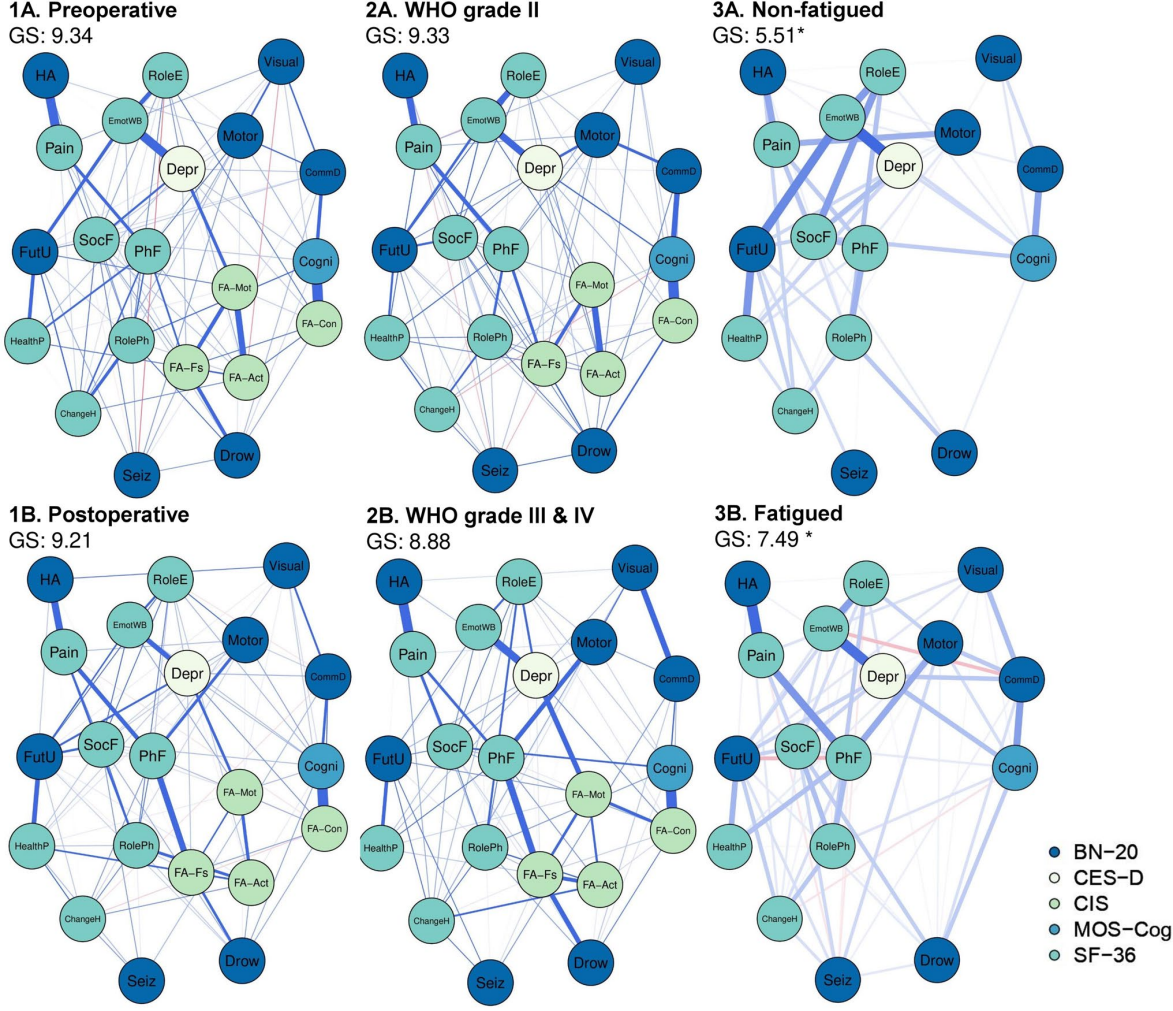
Symptom networks of the subgroups. Note that network 1A. Preoperative is identical to the network depicted in Fig. 2. *Statistically significant difference in global strength between the networks of the non-fatigued patients (subgroup 3A) compared to the fatigued patients (subgroup 3B). Abbreviations: GS, global strength; for abbreviations of the nodes and questionnaires, see Fig. 2

### Comparing symptom networks based on fatigue status

The symptom networks of the subgroups based on fatigue status are presented in Fig. 3A, B. Global strength of the non-fatigued network (subgroup 3A) was significantly lower than that of the fatigued network (subgroup 3B), indicating that symptoms were more tightly intercorrelated in fatigued patients (GS = 5.51 vs. GS = 7.49, *p* < 0.001). Again, edge weights of the networks were moderately correlated (*ρ* = 0.49, *p* < 0.001).

### Comparing global strength: sensitivity analyses

To test whether removing the four CIS nodes would also lead to differences in GS between the networks of other subgroups, we excluded the CIS nodes from the preoperative (1A) and postoperative (1B) networks, which did not result in a significant difference in GS (see Supplementary Figure S8).

There were 66 patients with a preoperative and postoperative assessment who were included in both subgroups 1A and 1B. GS was not different between the 1A and 1B networks after excluding these 66 patients from subgroup 1A (GS = 9.31 vs. GS = 9.21, *p* = 0.84). There were 33 patients with both a non-fatigued assessment and a fatigued assessment at another time point, who were thus included in both subgroups 3A and 3B. After excluding these 33 patients from subgroup 3B, GS was still statistically different between the 3A and 3B networks (GS = 5.51 vs. GS = 6.62, *p* = 0.01).

## Discussion

In the current study, we aimed to examine patterns of associations between patient-reported fatigue, depression, subjective cognitive complaints, brain tumor-related symptoms, and HRQoL by applying symptom network analysis. Additionally, we sought to compare symptom networks of subgroups of glioma patients based on disease characteristics and fatigue status. First, we showed that the 21 studied items and subscales of the questionnaires were highly intercorrelated and could be represented as a network. In particular, fatigue severity, depression, and social functioning were highly connected to other symptoms. Interestingly, we found that how tightly PROMs were connected did not differ between networks based on disease characteristics, but PROMs were more tightly intercorrelated in fatigued patients compared to non-fatigued patients.

In the presented networks, all nodes were connected to each other, signifying how symptoms and HRQoL are highly associated amongst each other. This was the case across all six networks, irrespective of clinical characteristics. Especially, the nodes reflecting fatigue severity, depression, and social functioning were highly connected to other nodes, which clinically implying that, in general, patients do not suffer from isolated symptoms, but instead from a very broad range of symptoms, complaints, and problems. These results add to our clinical understanding of the multidimensionality of symptoms and underline the importance of addressing, assessing, and treating symptoms as multidimensional, and not in isolation. In line with this is the finding that global strength did not differ between networks stratified based on disease characteristics, but global network strength was higher in fatigued patients in comparison to non-fatigued patients. This emphasizes that, importantly. Also, patient-reported variables, and not only clinical characteristics, determine which symptoms patients experience together, and to what extent.

These presented networks corroborate studies investigating the prevalence and burden of symptoms in glioma and are in line with studies identifying fatigue to be highly correlated to symptoms such as depression and physical functioning [1, 12, 13]. Comparably, studies on symptom networks in cancer patients have also found fatigue to be a central node in symptom networks [7, 10, 13, 14]. It has been hypothesized that these central symptoms may be suitable targets for therapeutic interventions, with successful treatment of a central symptom resulting in the simultaneous improvement of connected symptoms [41]. However, this theory has been debated, since such an effect would imply direct causality between symptoms and suggests that it should be possible to design an intervention that targets only one specific symptom, without addressing other symptoms [42, 43]. To date, experimental studies intervening on symptoms from a network perspective are lacking. With regard to the current study, it does stand out that fatigue plays an important role in glioma symptom networks. Unfortunately, there are no effective evidence-based treatments targeting fatigue in brain tumor patients, so developing integrative interventions targeting fatigue should be prioritized [44].

A similarly designed study in patients with gastric cancer before and after surgery and a second study in patients with head and neck cancer before and after radiotherapy showed that the global strength of symptom networks did not change over time [10, 45]. Interestingly, the study in patients with head and neck cancer found higher global strength in patients with higher stress levels [45]. Because global strength did not differ in networks based on disease characteristics, but did differ in networks based on fatigue status, the current results imply that symptom network density and the correlation between symptoms are not solely related to the disease itself, but that symptoms and HRQoL are also highly correlated amongst themselves [3]. To comprehend how network global strength relates to actual symptom burden in patients, it would be of value to investigate whether network global strength indeed decreases after successful symptom management in glioma patients.

As we have shown, all symptoms in the presented networks were highly correlated to each other. Applying symptom networks analysis, instead of more traditional statistical methods, is particularly useful when working with multivariate data, since it allows us to move past understanding or predicting single-outcome measures or symptoms [46]. However, it is important to emphasize that the associations between symptoms in the presented networks are correlative, and do not imply causality. An approach to address this gap would be to compute individual networks from high-density longitudinal individual data. By doing so, we can predict how symptoms influence each other over time [47]. Using this exact approach, a study in cancer survivors with depressive symptoms showed high scores on fatigue and worrying to be strongly predicted by their scores at the previous time point, suggesting self-reinforcing loops [48]. Additionally, individual-level symptom networks can be constructed with these data. In clinical practice, these individual networks have been used as part of psycho-education for fatigued cancer patients, with positive responses from patients [49]. Both these applications would be of great interest in glioma patients to improve our understanding of causal mechanisms behind the emergence of symptoms, and to guide psycho-education and symptom management in individual patients.

Several limitations should be taken into account when interpreting the presented symptom networks. Because the studied sample predominantly consisted of lower-grade tumors, we could not study the symptom network of glioblastoma patients separately [26]. Also, we did not take tumor characteristics like IDH status or tumor location into account, which could be interesting because of their link to functional status [50]. Furthermore, we did include the most common symptoms in glioma, like fatigue, subjective cognitive complaints, and depression, but we did not assess symptoms like anxiety, or chemotherapy-related symptoms. For future studies, we aim to assess a broader range of symptoms, which would also require a larger sample size [51]. Also, in the current study, the sample size of some of the subgroups was relatively small compared to other symptom network studies, reflected by the rather large confidence intervals of the edge weights. Another limitation is that some patients were represented in multiple subgroups as they contributed data on multiple occasions, for example preoperatively and postoperatively. Unfortunately, the paired version of the network comparison test is only available when the entire sample is paired, such as in a pre-post design [39]. Consequently, the assumption of independent observations is violated in the subgroup comparisons. To investigate whether this biased our results, we excluded patients that were represented in multiple subgroups and compared GS between the networks of these groups. The results were similar, namely a significant difference in GS between the non-fatigued and fatigued subgroups. Lastly, the chosen regularization technique affects the number of presented edges in the networks. Because of the exploratory nature of the study, we did not apply a very strict regularization method, since this would result in less spurious edges, but could also result in the removal of true edges [4].

In conclusion, symptom network analysis is a novel approach to uncover the complexity of symptom interactions in glioma. Symptom networks might be specifically valuable in guiding symptom management, finding relevant treatment targets, and personalizing treatment. Interestingly, in this study, we showed that symptom networks in glioma did not differ according to disease status and tumor grade, while we did find that PROMs were more tightly intercorrelated in patients suffering from fatigue. This underlines the need for integrative symptom management targeting fatigue. Our findings add to a growing body of literature underlining how symptoms are not only caused by the disease itself, but are also highly correlated amongst themselves.

### Supplementary Information

Below is the link to the electronic supplementary material.

Supplementary file1 (PDF 1.66 MB)

## Data Availability

Raw data cannot be shared because of privacy reasons. Therefore, we have generated comparable synthetic data. The scripts for the statistical analyses are available in an online repository (https://github.com/multinetlab-amsterdam/projects/tree/master/symptomnetwork_glioma_paper_2022) and can be run with the provided synthetic data.

## Abbreviations

*BN-20*: European Organization for Research and Treatment of Cancer brain tumor module
*CES-D*: Center for Epidemiologic Studies Depression
*CIS*: Checklist Individual Strength
*EBICglasso*: Extended Bayesian information criterion with least absolute shrinkage and selection operator
*GS*: Global strength
*HRQoL*: Health-related quality of life
*LASSO*: Least absolute shrinkage and selection operator
*IDH*: Isocitrate dehydrogenase
*MOS-Cog*: Medical Outcomes Study Cognitive Functioning Scale
*SF-36*: Medical Outcomes Study Short-Form Health Survey
*WHO*: World Health Organization
