# Supplementary material for: Symptom networks in glioma patients: understanding the multidimensionality of symptoms and quality of life
Source: J Cancer Surviv. 2023 Mar 16;18(3):1032–41. doi: 10.1007/s11764-023-01355-8 (PMC11082018; doi:10.1007/s11764-023-01355-8)
Supplement: Supplementary file 1 — Supplementary file1 (PDF 1.66 MB) [file 11764_2023_1355_MOESM1_ESM.pdf]

Supplementary materials

Symptom networks in glioma patients: understanding the multidimensionality of symptoms and quality of life

J.G. Röttgering[1,2]*, T.M.C.K. Varkevisser[3], M. Gorter[1,3], V. Belgers[1,4], P. de Witt Hamer[1,5], J.C. Reijneveld[6], M. Klein[1,2], T.F. Blanken[7], L. Douw[1,3]

[1] Cancer Center Amsterdam, Brain Tumor Center, Amsterdam, The Netherlands
[2] Amsterdam UMC location Vrije Universiteit Amsterdam, Medical Psychology, Boelelaan 1117, Amsterdam, The Netherlands
[3] Amsterdam UMC location Vrije Universiteit Amsterdam, Anatomy and Neurosciences, Boelelaan 1117, Amsterdam, The Netherlands
[4] Amsterdam UMC location Vrije Universiteit Amsterdam, Neurology, Boelelaan 1117, Amsterdam, The Netherlands
[5] Amsterdam UMC location Vrije Universiteit Amsterdam, Neurosurgery, Boelelaan 1117, Amsterdam, The Netherlands
[5] Amsterdam Public Health Research Institute, Amsterdam, The Netherlands
[6] Department of Neurology, SEIN, Heemstede, The Netherlands
[7] Department of Psychological Methods, University of Amsterdam, 1018 WT, Amsterdam, the Netherlands
* Corresponding author, j.rottgering@amsterdamumc.nl

Table S1. Overview of publications using the presented data 3

Table S2. Nodes and corresponding questionnaire subscales 4

Figure S1. Flowchart of the selection of patients, based on the three research questions 5

Edge-weight accuracy and node strength stability 6

Table S3. Outcome of the questionnaires per subgroup 7

Table S4. Regularized partial correlation matrix of the preoperative network (subgroup 1A) 8

Figure S3. Node strength and stability indices of the postoperative network (subgroup 1B) 11

Figure S4. Node strength and stability indices of the grade II tumor network (subgroup 2A) 12

Figure S5. Node strength and stability indices of the grade III/IV tumor network (subgroup 2B) ... 13

Figure S6. Node strength and stability indices of the non-fatigue network (subgroup 3A) 14

Figure S7. Node strength and stability indices of the fatigue network (subgroup 3B) 15

Figure S8. Difference in GS between preoperative and postoperative networks excluding fatigue nodes 16

References supplementary materials 17

**Author, year, reference**	**Title**	**Study design**	**Primary outcome measure**	**Included variables**	**Included patients**
Douw et al. 2010 [1]	Epilepsy is related to theta band brain connectivity and network topology in brain tumor patients	Prospective longitudinal observational study	MEG functional connectivity and network topology measures	MEG preop and postop	17 diffuse glioma patients
van Dellen et al.2012 [2]	MEG Network Differences between Low- and High-Grade Glioma Related to Epilepsy and Cognition	Retrospective cross-sectional observational study	MEG functional connectivity and network topology measures	MEG, NPA preop	35 patients:<br>13 low-grade glioma<br>12 high grade glioma<br>10 non-glioma
van Dellen et al. 2012 [3]	Connectivity in MEG resting-state networks increases after resective surgery for low-grade glioma and correlates with improved cognitive performance.	Prospective longitudinal observational study	MEG functional connectivity and cognitive measures	MEG and NPA preop and postop	10 low grade glioma patients
Carbo et al. 2017 [4]	Dynamic hub load predicts cognitive decline after resective neurosurgery	Retrospective longitudinal observational study	MEG hub load score and cognitive measures	MEG and NPA preop and postop	28 patients of which 21 diffuse glioma patients
Derks et al.2018 [5]	Oscillatory brain activity associates with neuroligin-3 expression and predicts progression free survival in patients with diffuse glioma.	Retrospective cross-sectional observational study	MEG broadband power and neuroligin-3 expression	MEG preop, immunohistochemistry	24 diffuse glioma patients
Derks et al.2019 [6]	Understanding cognitive functioning in glioma patients: The relevance of IDH-mutation status and functional connectivity	Retrospective cross-sectional observational study	MEG functional connectivity and IDH status	MEG,NPA preop and immunohistochemistry	54 diffuse glioma patients
Belgers et al.2020 [7]	Postoperative oscillatory brain activity as an add-on prognostic marker in diffuse glioma.	Retrospective cross-sectional observational study	MEG broadband power and progression-free survival	MEG preop	27 diffuse glioma patients
Numan et al.2021 [8]	Non-invasively measured brain activity and radiological progression in diffuse glioma	Retrospective cross-sectional observational study	MEG brain activity and MRI tumor progression	MEG postop	45 diffuse glioma patients

Derks et al.2021 [9]	Understanding Global Brain Network Alterations in Glioma Patients	Retrospective cross-sectional observational studys	MEG network clustering and MRI tumor maps	MEG preop	71 diffuse glioma patients
Röttgering et al. 2022 [10]	Toward unraveling the correlates of fatigue in glioma	Retrospective longitudinal observational study	Checklist individual strength, subscale fatigue severity	Questionnaires preop and postop	222 glioma patients

Table S1. Overview of publications using the presented data

Abbreviations: MEG, magnetoencefalography; NPA, neuropsychological assessment; preop, preoperatively; postop, postoperatively;

Node	Validated subscale	Questionnaire	Items from questionnaire
FA-Fs	CIS fatigue severity	Checklist Individual Strength (CIS) [11]	1, 4, 6, 9, 12, 14, 16, 20
FA-Con	CIS concentration problems		3, 8, 11, 13, 19
FA-Mot	CIS reduced motivation		2, 5, 15, 18
FA-Act	CIS reduced activity level		7, 10, 17
Depr	-	Center for Epidemiologic Studies Depression questionnaire (CES-D) [12]	All 20 items
Cogni	-	Medical Outcomes Study Cognitive Functioning Scale (MOS-Cog) [13]	All 6 items
FutU	BN-20 future uncertainty	European Organization for Research and Treatment of Cancer brain tumor module (BN-20) [14]	31, 32, 33, 35
Visual	BN-20 visual disorder		36, 37, 38
Motor	BN-20 motor dysfunction		40, 45, 49
CommD	BN-20 communication deficit		41, 42, 43
HA	BN-20 headaches		34
Seiz	BN-20 seizures		39
Drow	BN-20 drowsiness		44
PhF	SF-36 Physical functioning	36-Item Short Form Survey (SF-36) [15]	3, 4, 5, 6, 7, 8, 9, 10, 11, 12
SocF	SF-36 Social functioning		20, 32
RolePh	SF-36 Role limitations due to physical health		13, 14, 15, 16
RoleE	SF-36 Role limitations due to emotional problems		17, 18, 19
EmotWB	SF-36 Emotional well-being		24, 25, 26, 28, 30
Pain	SF-36 Pain		21, 22
HealthP	SF-36 General health perception		1, 33, 34, 35, 36
ChangeH	SF-36 Change in health		2

Table S2. Nodes and corresponding questionnaire subscales

Each node in the networks corresponds to a questionnaire or a validated subscale of a questionnaire.

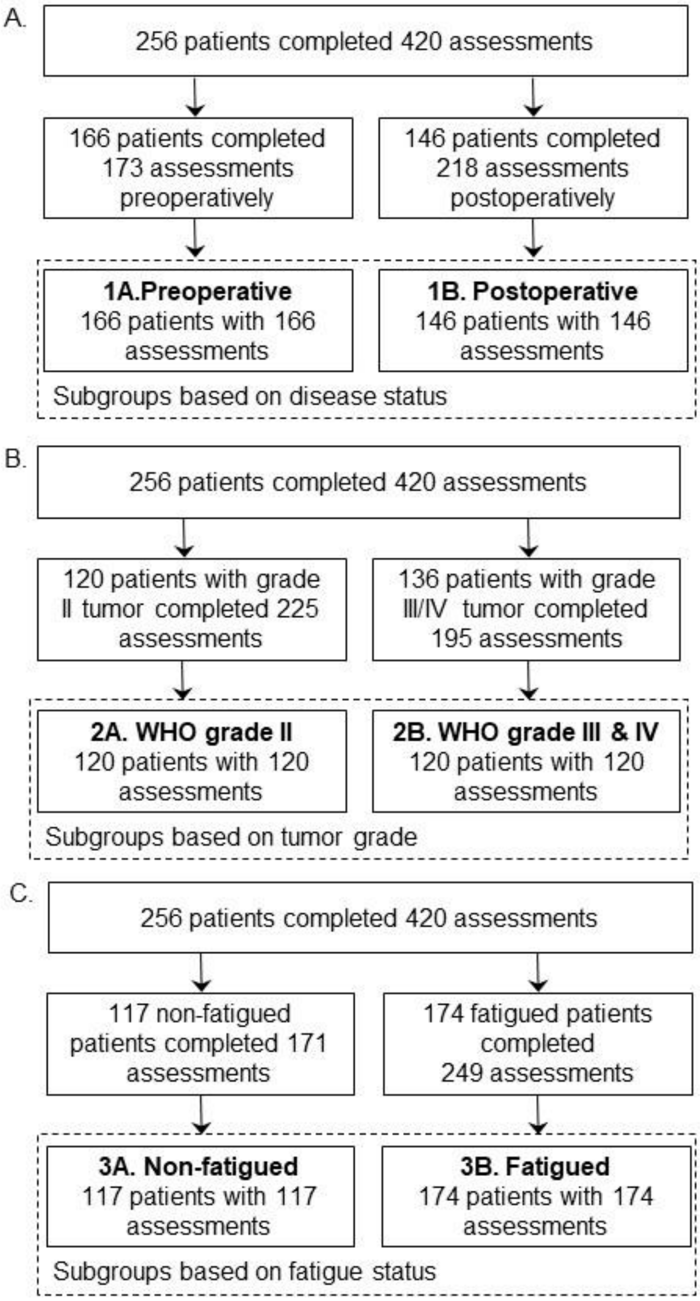

Figure S1. Flowchart of the selection of patients, based on the three research questions
For each of the research questions, the entire cohort of patients was divided into two subgroups based on either 1) disease status, 2) tumor grade, and 3) fatigue status. Then, one measurement per patient was selected at random, to avoid within-subject duplicates.

A. Selection of patients based on disease status (subgroups 1A and 1B)
B. Selection of patients based on tumor grade (subgroups 2A and 2B)
C. Selection of patients based on fatigue status (subgroups 4A and 3B)

Abbreviations: WHO, World Health Organization.

Edge-weight accuracy and node strength stability

In the presented networks, each of the potential edges was estimated with Spearman's partial correlations. For a network with five nodes, 5x4/2=10 edges need to be estimated, and for a network with 21 nodes, 21×20/2= 210. Because of the large number of estimated parameters in the presented networks, it is important to quantify the accuracy of the estimated edges and the stability of the calculated node strengths [16, 17]. The plots of these accuracy and stability analyses are presented below (Figure S2-S7).

To assess edge-weight accuracy, we estimated a 95% confidence interval of the edge weights for each network, based on bootstrapping with 1000 iterations [16]. Larger confidence intervals indicate low precision of the estimated edge weight. Indeed, as can be seen from the confidence intervals around the estimated edge weights, there is quite some uncertainty around the estimated edge weights. This indicates that the exact weight of the estimated edges should be interpreted with some caution.

To assess the stability of the strength of the nodes, we performed a case-dropping bootstrap with 1000 iterations and computed a correlation stability coefficient (CS-coefficient) using Bootnet (version 1.4.3) [16]. The CS-coefficient quantifies the maximum proportion of cases that can be dropped at random to retain a correlation of at least 0.7 with the nodal strength values of the original network. This value should preferably be ≥ 0.5, but at least ≥ 0.25. As can be seen from the computed CS-coefficients, all CS-coefficients are larger than the minimum cut-off of 0.25. The CS-coefficient of the networks in subgroups 1A and 1B are above the cut-off of 0.5, indicating higher stability of the computed strength centrality of the nodes.

	1A. Preoperative (N=166)	1B. Postoperative (N=146)	2A. Grade II (N=120)	2B. Grade III & IV (N=136)	3A. Non-fatigued (N=117)	3B. Fatigued (N=174)
**CIS subjective fatigue**	28.5 [19.0, 37.8]	32.0 [20.0, 41.8]	29.5 [17.8, 39.0]	30.0 [20.0, 41.0]	16.0 [11.0, 22.0]	36.0 [31.0, 44.0]
**CIS concentration**	15.0 [10.0, 22.0]	18.0 [11.0, 25.0]	15.0 [11.0, 24.3]	16.0 [9.75, 22.0]	11.0 [7.00, 15.0]	20.0 [13.3, 25.0]
**CIS motivation**	12.0 [8.00, 17.8]	14.0 [8.25, 18.0]	12.0 [7.75, 17.0]	14.0 [9.75, 19.0]	8.00 [5.00, 11.0]	16.0 [12.0, 19.0]
**CIS activity**	10.0 [6.00, 15.0]	11.0 [7.00, 15.8]	10.0 [6.00, 14.3]	12.0 [7.00, 16.3]	7.00 [4.00, 10.0]	13.0 [9.00, 17.0]
**CES-D score depression**	11.0 [6.00, 16.0]	11.0 [4.25, 18.0]	10.0 [5.00, 17.0]	11.5 [7.00, 17.0]	6.00 [2.00, 10.0]	13.0 [10.0, 20.0]
**MOS-Cog**	12.0 [9.00, 17.0]	15.5 [10.0, 20.0]	13.0 [9.00, 18.0]	13.0 [9.00, 17.0]	11.0 [8.00, 14.0]	16.0 [11.0, 19.0]
**BN-20 future uncertainty**	41.7 [25.0, 64.6]	33.3 [16.7, 64.6]	33.3 [25.0, 58.3]	41.7 [25.0, 58.3]	25.0 [8.33, 50.0]	50.0 [33.3, 66.7]
**BN-20 visual disorder**	0 [0, 22.2]	0 [0, 22.2]	0 [0, 22.2]	0 [0, 22.2]	0 [0, 11.1]	11.1 [0, 22.2]
**BN-20 motor dysfunction**	0 [0, 22.2]	11.1 [0, 22.2]	0 [0, 22.2]	11.1 [0, 22.2]	0 [0, 11.1]	11.1 [0, 22.2]
**BN-20 communication deficit**	16.7 [0, 33.3]	22.2 [0, 33.3]	11.1 [0, 33.3]	22.2 [0, 33.3]	11.1 [0, 22.2]	22.2 [0, 41.7]
**BN-20 headaches**	33.3 [0, 33.3]	0 [0, 33.3]	33.3 [0, 33.3]	33.3 [0, 33.3]	0 [0, 33.3]	33.3 [0, 33.3]
**BN-20 seizures**	0 [0, 33.3]	0 [0, 25.0]	0 [0, 33.3]	0 [0, 33.3]	0 [0, 0]	0 [0, 33.3]
**BN-20 drowsiness**	33.3 [0, 33.3]	33.3 [0, 33.3]	33.3 [0, 33.3]	33.3 [0, 33.3]	0 [0, 33.3]	33.3 [33.3, 66.7]
**SF-36 Physical functioning**	10.0 [0, 20.0]	10.0 [5.00, 35.0]	5.00 [0, 20.0]	10.0 [5.00, 35.0]	0 [0, 5.00]	20.0 [10.0, 38.8]
**SF-36 Social functioning**	25.0 [12.5, 50.0]	37.5 [12.5, 50.0]	25.0 [0, 50.0]	37.5 [12.5, 50.0]	12.5 [0, 25.0]	37.5 [25.0, 62.5]
**SF-36 Role limitation - Physical**	62.5 [0, 100]	75.0 [0, 100]	50.0 [0, 100]	75.0 [0, 100]	0 [0, 50.0]	100 [50.0, 100]
**SF-36 Role limitation - Emotional**	16.7 [0, 100]	33.3 [0, 66.7]	0 [0, 66.7]	33.3 [0, 100]	0 [0, 33.3]	66.7 [0, 100]
**SF-36 Mental Health**	28.0 [16.0, 47.0]	28.0 [16.0, 48.0]	28.0 [16.0, 45.0]	32.0 [20.0, 44.0]	20.0 [12.0, 32.0]	36.0 [24.0, 48.0]
**SF-36 Bodily pain**	10.2 [0, 32.7]	20.4 [0, 42.9]	10.2 [0, 32.7]	12.2 [0, 32.7]	0 [0, 12.2]	22.4 [0, 44.9]
**SF-36 General health perceptions**	40.0 [26.3, 55.0]	50.0 [30.0, 65.0]	45.0 [30.0, 60.0]	45.0 [30.0, 60.0]	30.0 [20.0, 45.0]	55.0 [36.3, 65.0]
**SF-36 Change in health**	75.0 [50.0, 75.0]	50.0 [25.0, 75.0]	50.0 [25.0, 75.0]	75.0 [50.0, 100]	50.0 [25.0, 50.0]	75.0 [50.0, 100]

Table S3. Outcome of the questionnaires per subgroup
All values are presented as median [interquartile range].

*Abbreviations: **BN-20**, European Organization for Research and Treatment of Cancer brain tumor module; **CES-D**, depressive symptoms**;** **CIS**, Checklist Individual Strength; **MOS-Cog** subjective cognitive complaints; **SF-36,** 36-Item Short Form Survey.*

	FA-Fs	FA-Con	FA-Mot	FA-Act	Cogni	Depr	PhF	SocF	RolePh	RoleE	EmotWB	Pain	HealthP	ChangeH	FutU	Visual	Motor	CommD	HA	Seiz	Drow
FA-Fs		0.1228	0.227	0.1493	0	0.0638	0.1572	0.0778	0	0	0	0.0394	0.1185	0	0	0	0	0	0	0.0756	0.2169
FA-Con			0.0439	0.125	0.4395	0.0484	0	0.0053	0	0.1168	0	0	0	0	0	0.0476	0	0.0037	0	0	0.0779
FA-Mot				0.3017	0	0.203	0	0	0	0	0	0	0.0182	0	0.1148	0	0	0	0	0	0
FA-Act					0	0.0131	0.0684	0.0551	0.0449	0.0683	0	0.0019	0	0.1021	0	-0.056	0	0	0	0	0
Cogni						0.055	0	0.0523	0.1342	0	0	0	0	0	0	0	0.0676	0.2088	0	0	0.0362
Depr							0	0.1666	0	0.094	0.391	0	0.0575	0	0	0.0404	0.002	0.0541	0.003	0	0
PhF								0.0425	0.1443	0	0	0.2005	0.1606	0.1498	0	0.0236	0.1236	0	0	0	0
SocF									0.0949	0.0793	0.0466	0.1147	0	0.1201	0.1063	0	0.0475	0.046	0	0.1089	0.0479
RolePh										0.1075	0	0	0	0.2002	0.0427	0	0.0604	0	0.089	0	0.1284
RoleE											0.2432	0	0	0.0263	0.0249	0	0.0202	0	0	-0.073	0
EmotWB												0	0	0	0.2082	0	0	0	0	0.062	0
Pain													0.0036	0.0324	0	0.0757	0	0	0.3993	0	0.0137
HealthP														0.0042	0.2161	0	0	0	0	0	0
ChangeH															0.1391	0	0.0758	0	0	0	0
FutU																0	0.021	0.0304	0	0.0756	0
Visual																	0.1582	0.1482	0	0	0.0055
Motor																		0.1394	0.046	0.09	0.0242
CommD																			0	0.0207	0.0653
HA																				-0.01	0
Seiz																					0.0859
Drow																					

Table S4. Regularized partial correlation matrix of the preoperative network (subgroup 1A)

This is the regularized partial correlation matrix that is used as input for the visualization of the symptom network of the preoperative patients (subgroup 1A). Each number is the equivalent of an edge between two symptoms. If a number is zero, then there is no edge between two symptoms. A blue number indicates a

positive relation and a red number a negative relation. For example, CIS-F and CIS-C are connected by an edge with an edge weight of 0.1228. There are 98 edges present in this matrix/network.

Abbreviations:
BN-20, European Organization for Research and Treatment of Cancer brain tumor module; **ChangeH**, SF-36 Change in health; **CIS**, Checklist Individual Strength; **Cogni**, Medical Outcomes Study Cognitive Functioning Scale (MOS-Cog); **CommD**, BN-20 communication deficit; **Depr**, Center for Epidemiologic Studies Depression questionnaire (CES-D); **Drow**, BN-20 drowsiness; **EmotWB**, SF-36 Emotional well-being; **FA-Act**, *CIS* reduced activity level ; **FA-con**, *CIS* concentration problems; ***FA-Fs***, *CIS* fatigue severity; **FA-Mot**, CIS reduced motivation; **FutU**, BN-20 future uncertainty; **HA**, BN-20 headaches; **HealthP**, SF-36 General health perception; **Motor**, BN-20 motor dysfunction; **Pain**, SF-36 Pain; **PhF**, *SF-36 Physical functioning;* **RoleE**, SF-36 Role limitations due to emotional problems; **RolePh**, SF-36 Role limitations due to physical health; **Seiz**, BN-20 seizures;**SF-36**, 36-Item Short Form Survey; **SocF**, SF-36 Social functioning; **Visual**, BN-20 visual disorder;

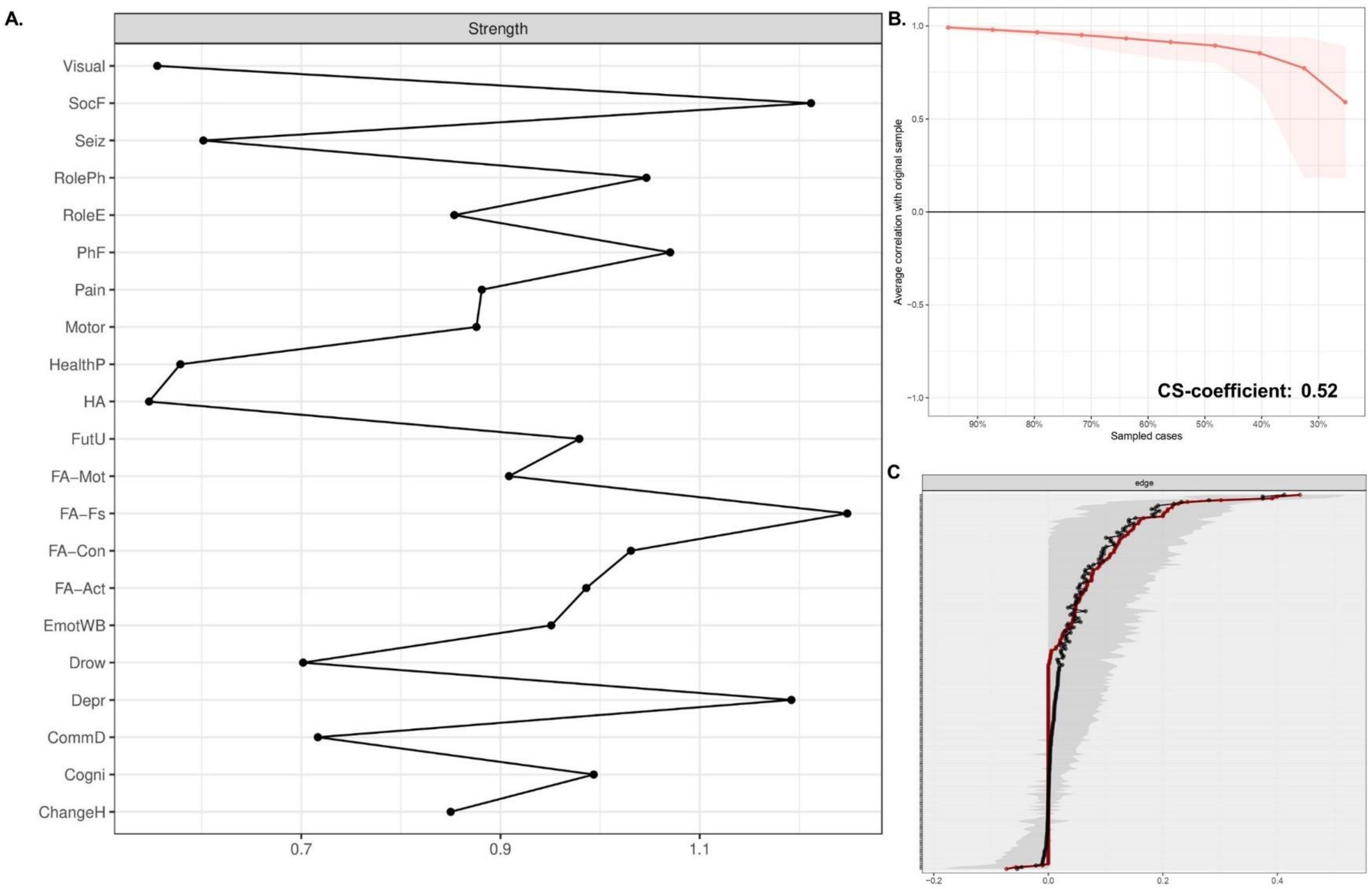

Figure S2. Node strength and stability indices of the preoperative network (subgroup 1A)

A. Node strength

The y-axis shows all 21 nodes. The x-axis shows the node strength.

B. Case-dropping bootstrap.

The x-axis shows the percentage of sampled cases dropped which decreases by 10% each time. The y-axis shows the average correlation of the centrality stability coefficient with the original sample.

C. Bootstrapped 95% confidence intervals of the edge weights.

The y-axis shows all edges in the network ordered from the largest to smallest from top to bottom. The x-axis shows the confidence interval range. The red line represents the edge weights of the network, and the grey bars indicate the 95% CIs around the edge weights.

*Abbreviations: **BN-20**, European Organization for Research and Treatment of Cancer brain tumor module; **ChangeH**, SF-36 Change in health; **CIS**, Checklist Individual Strength; **Cogni**, Medical Outcomes Study Cognitive Functioning Scale (MOS-Cog); **CommD**, BN-20 communication deficit; **Depr**, Center for Epidemiologic Studies Depression questionnaire (CES-D); **Drow**, BN-20 drowsiness; **EmotWB**, SF-36 Emotional well-being; **FA-Act**, CIS reduced activity level ; **FA-con**, CIS concentration problems; **FA-Fs**, CIS fatigue severity; **FA-Mot**, CIS reduced motivation; **FutU**, BN-20 future uncertainty; **HA**, BN-20 headaches; **HealthP**, SF-36 General health perception; **Motor**, BN-20 motor dysfunction; **Pain**, SF-36 Pain; **PhF**, SF-36 Physical functioning; **RoleE**, SF-36 Role limitations due to emotional problems;**RolePh**, SF-36 Role limitations due to physical health; **Seiz**, BN-20 seizures;**SF-36**, 36-Item Short Form Survey; **SocF**, SF-36 Social functioning; **Visual**, BN-20 visual disorder;*

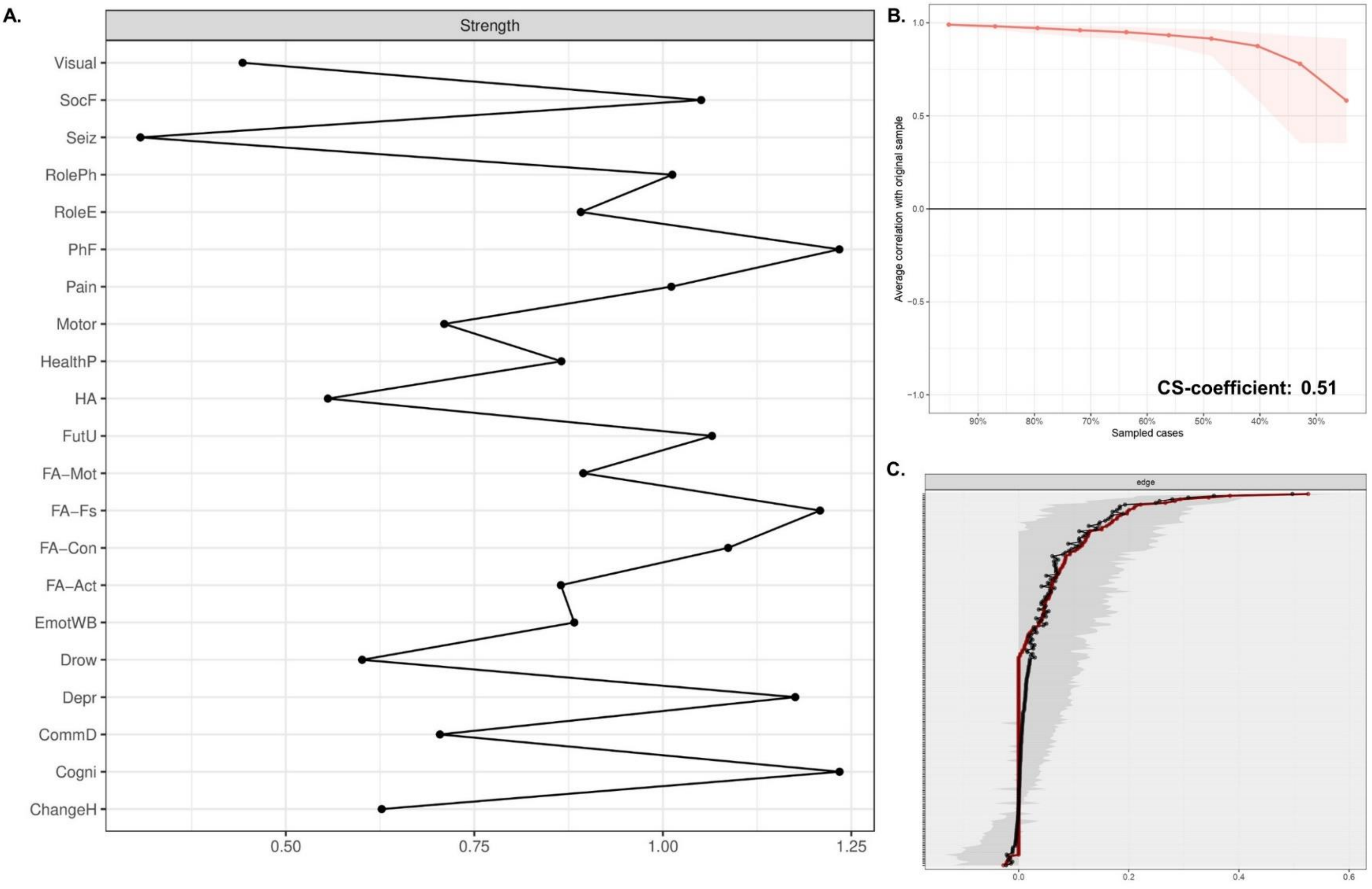

Figure S3. Node strength and stability indices of the postoperative network (subgroup 1B)

A. Node strength

The y-axis shows all 21 nodes. The x-axis shows the node strength.

B. Case-dropping bootstrap.

The x-axis shows the percentage of sampled cases dropped which decreases by 10% each time. The y-axis shows the average correlation of the centrality stability coefficient with the original sample.

C. Bootstrapped 95% confidence intervals of the edge weights.

The y-axis shows all edges in the network ordered from the largest to smallest from top to bottom. The x-axis shows the confidence interval range. The red line represents the edge weights of the network, and the grey bars indicate the 95% CIs around the edge weights.

Abbreviations: see figure S2

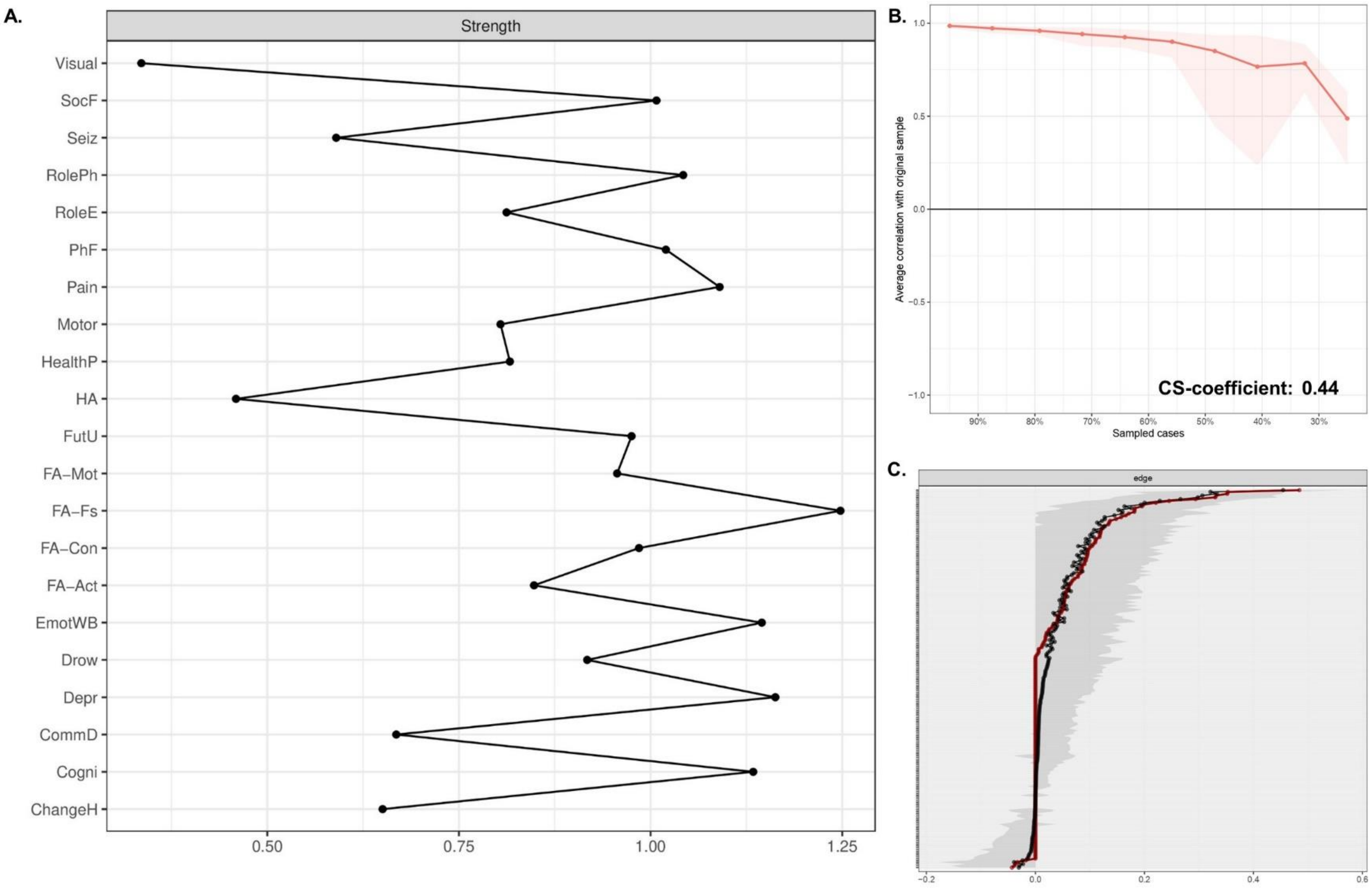

Figure S4. Node strength and stability indices of the grade II tumor network (subgroup 2A)

A. Node strength

The y-axis shows all 21 nodes. The x-axis shows the node strength.

B. Case-dropping bootstrap.

The x-axis shows the percentage of sampled cases dropped which decreases by 10% each time. The y-axis shows the average correlation of the centrality stability coefficient with the original sample.

C. Bootstrapped 95% confidence intervals of the edge weights.

The y-axis shows all edges in the network ordered from the largest to smallest from top to bottom. The x-axis shows the confidence interval range. The red line represents the edge weights of the network, and the grey bars indicate the 95% CIs around the edge weights.

Abbreviations: see figure S2

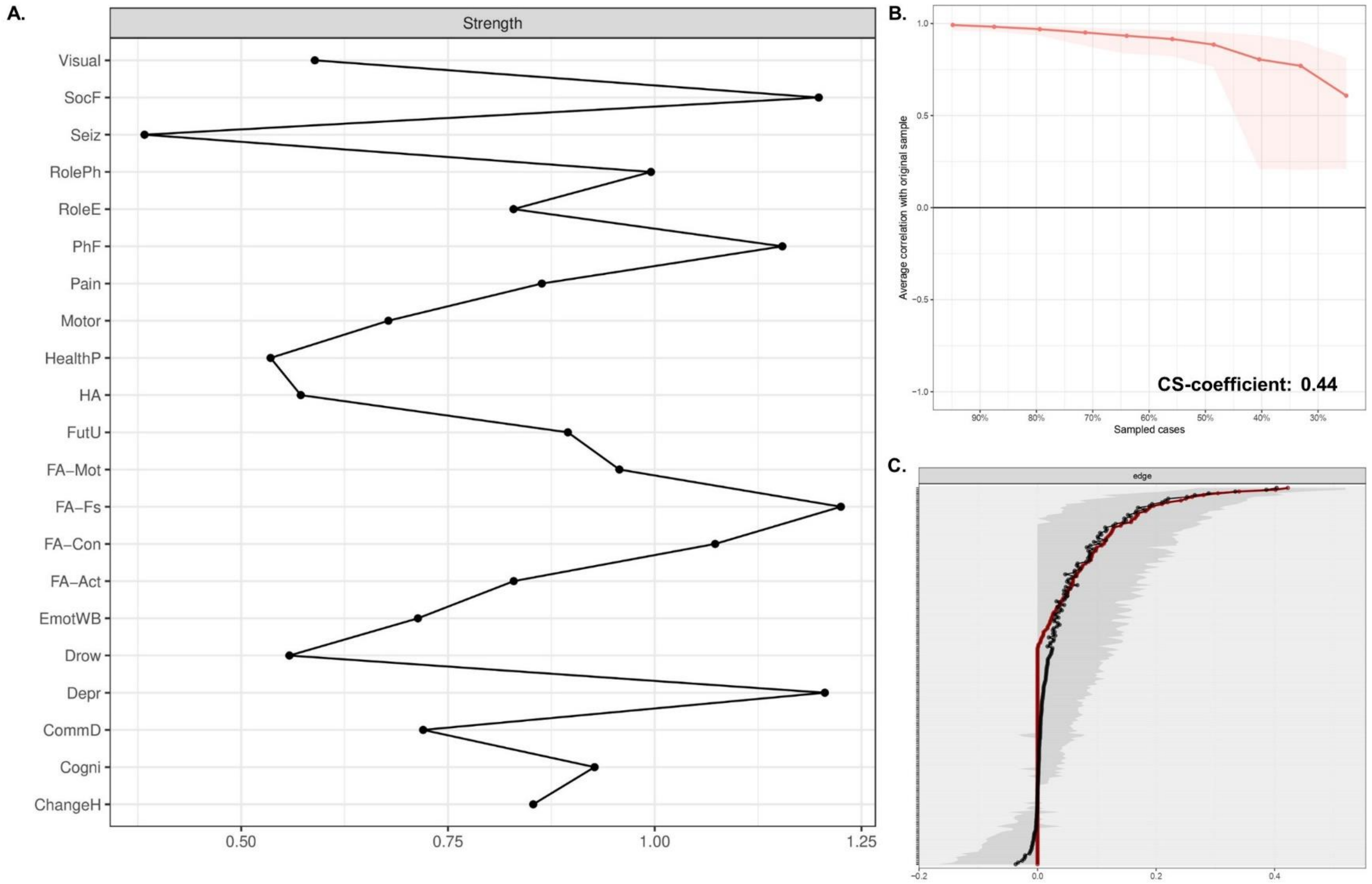

Figure S5. Node strength and stability indices of the grade III/IV tumor network (subgroup 2B)

A. Node strength

The y-axis shows all 21 nodes. The x-axis shows the node strength.

B. Case-dropping bootstrap.

The x-axis shows the percentage of sampled cases dropped which decreases by 10% each time. The y-axis shows the average correlation of the centrality stability coefficient with the original sample.

C. Bootstrapped 95% confidence intervals of the edge weights.

The y-axis shows all edges in the network ordered from the largest to smallest from top to bottom. The x-axis shows the confidence interval range. The red line represents the edge weights of the network, and the grey bars indicate the 95% CIs around the edge weights.

Abbreviations: see figure S2

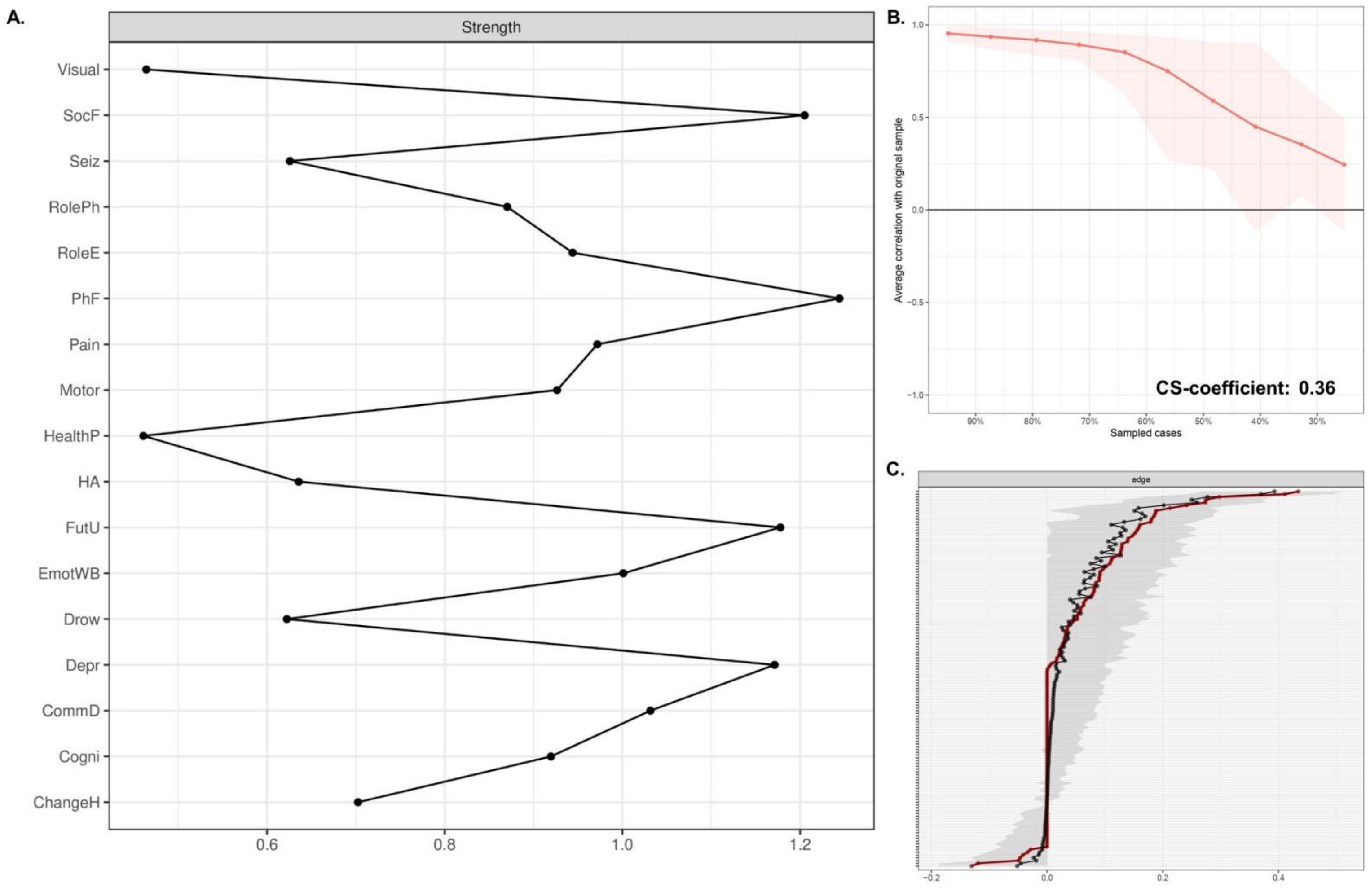

Figure S6. Node strength and stability indices of the non-fatigue network (subgroup 3A)

A. Node strength

The y-axis shows all 21 nodes. The x-axis shows the node strength.

B. Case-dropping bootstrap.

The x-axis shows the percentage of sampled cases dropped which decreases by 10% each time. The y-axis shows the average correlation of the centrality stability coefficient with the original sample.

C. Bootstrapped 95% confidence intervals of the edge weights.

The y-axis shows all edges in the network ordered from the largest to smallest from top to bottom. The x-axis shows the confidence interval range. The red line represents the edge weights of the network, and the grey bars indicate the 95% CIs around the edge weights.

Abbreviations: see figure S2

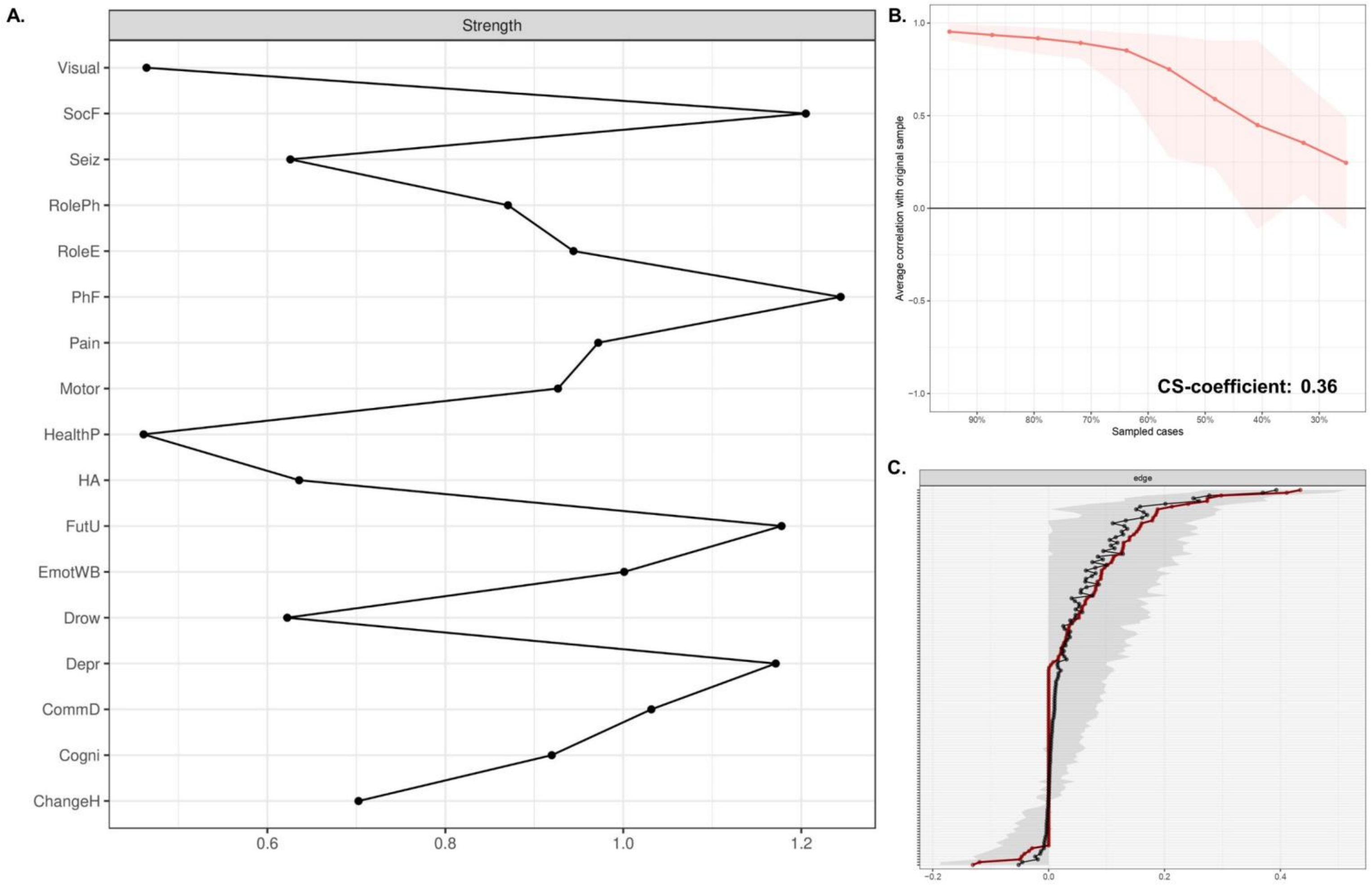

Figure S7. Node strength and stability indices of the fatigue network (subgroup 3B)

A. Node strength

The y-axis shows all 21 nodes. The x-axis shows the node strength.

B. Case-dropping bootstrap.

The x-axis shows the percentage of sampled cases dropped which decreases by 10% each time. The y-axis shows the average correlation of the centrality stability coefficient with the original sample.

C. Bootstrapped 95% confidence intervals of the edge weights.

The y-axis shows all edges in the network ordered from the largest to smallest from top to bottom. The x-axis shows the confidence interval range. The red line represents the edge weights of the network, and the grey bars indicate the 95% CIs around the edge weights.

Abbreviations: see figure S2

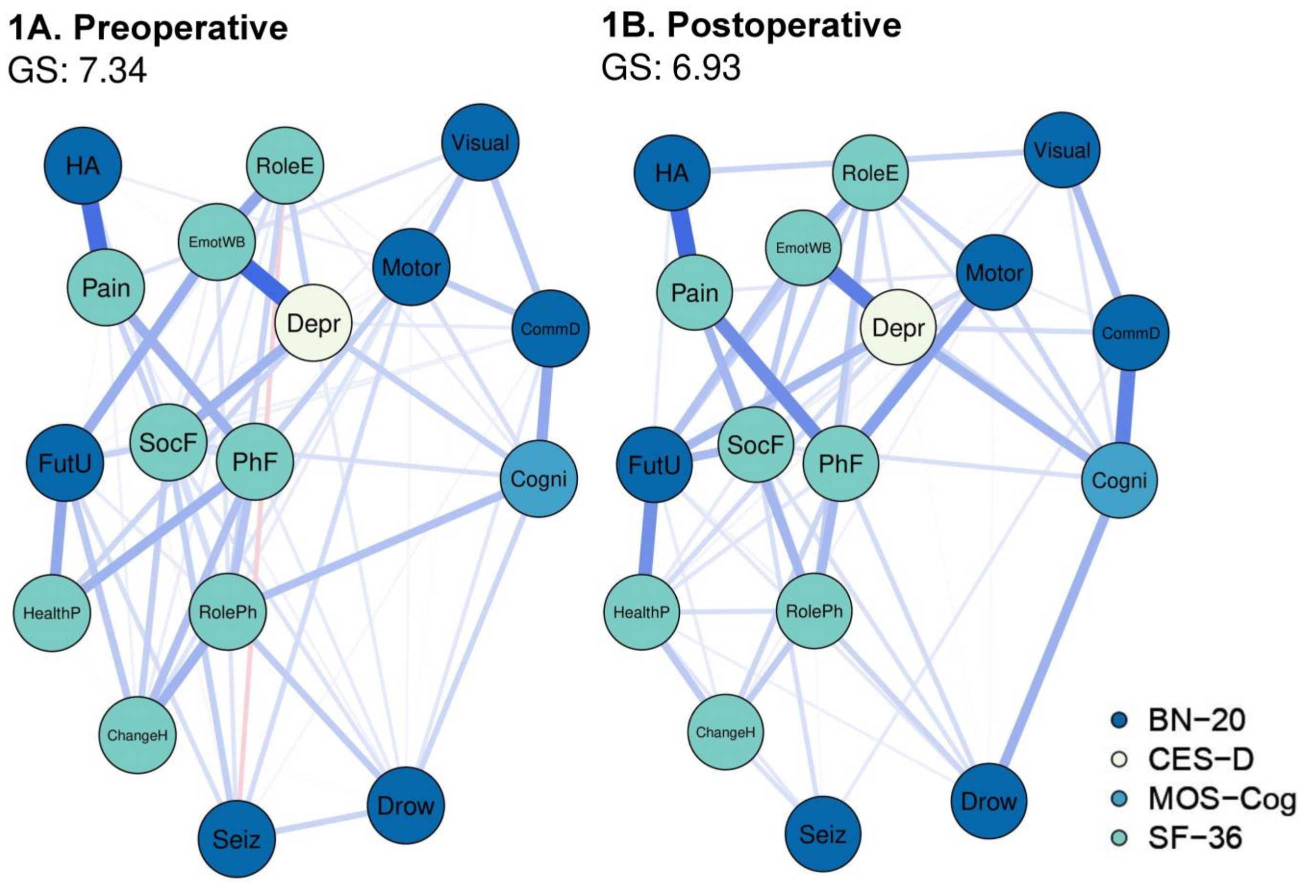

Figure S8. Difference in GS between preoperative and postoperative networks excluding fatigue nodes

After excluding the CIS nodes, there was no difference in global strength (GS 7.34 versus GS 6.93, p=0.21) between the networks of the preoperative and postoperative subgroups.

Abbreviations:

***BN-20**, European Organization for Research and Treatment of Cancer brain tumor module; **ChangeH**, SF-36 Change in health; **CIS**, Checklist Individual Strength; **Cogni**, Medical Outcomes Study Cognitive Functioning Scale (MOS-Cog); **CommD**, BN-20 communication deficit; **Depr**, Center for Epidemiologic Studies Depression questionnaire (CES-D); **Drow**, BN-20 drowsiness; **EmotWB**, SF-36 Emotional well-being; **FutU**, BN-20 future uncertainty; **HA**, BN-20 headaches; **HealthP**, SF-36 General health perception; **Motor**, BN-20 motor dysfunction; **Pain**, SF-36 Pain; **PhF**, SF-36 Physical functioning; **RoleE**, SF-36 Role limitations due to emotional problems; **RolePh**, SF-36 Role limitations due to physical health; **Seiz**, BN-20 seizures;**SF-36**, 36-Item Short Form Survey; **SocF**, SF-36 Social functioning; **Visual**, BN-20 visual disorder;*

References supplementary materials

1. Douw, L., et al., *Epilepsy is related to theta band brain connectivity and network topology in brain tumor patients.* BMC Neurosci, 2010. **11**: p. 103.
2. van Dellen, E., et al., *MEG Network Differences between Low- and High-Grade Glioma Related to Epilepsy and Cognition.* PLoS ONE, 2012. **7**.
3. van Dellen, E., et al., *Connectivity in MEG resting-state networks increases after resective surgery for low-grade glioma and correlates with improved cognitive performance.* Neuroimage Clin, 2012. **2**: p. 1-7.
4. Carbo, E.W.S., et al., *Dynamic hub load predicts cognitive decline after resective neurosurgery.* Scientific Reports, 2017. **7**(1): p. 42117.
5. Derks, J., et al., *Oscillatory brain activity associates with neuroligin-3 expression and predicts progression free survival in patients with diffuse glioma.* J Neurooncol, 2018. **140**(2): p. 403-412.
6. Derks, J., et al., *Understanding cognitive functioning in glioma patients: The relevance of IDH-mutation status and functional connectivity.* Brain Behav, 2019. **9**(4): p. e01204.
7. Belgers, V., et al., *Postoperative oscillatory brain activity as an add-on prognostic marker in diffuse glioma.* J Neurooncol, 2020. **147**(1): p. 49-58.
8. Numan, T., et al., *Non-invasively measured brain activity and radiological progression in diffuse glioma.* Scientific Reports, 2021. **11**(1): p. 18990.
9. Derks, J., et al., *Understanding Global Brain Network Alterations in Glioma Patients.* Brain Connect, 2021. **11**(10): p. 865-874.
10. Röttgering, J.G., et al., *Toward unraveling the correlates of fatigue in glioma.* Neuro-Oncology Advances, 2022.
11. Worm-Smeitink, M., et al., *The assessment of fatigue: Psychometric qualities and norms for the Checklist individual strength.* J Psychosom Res, 2017. **98**: p. 40-46.
12. Radloff, L.S., *The CES-D Scale:A Self-Report Depression Scale for Research in the General Population.* Applied Psychological Measurement, 1977. **1**(3): p. 385-401.
13. Stewart A, W.J., Jr, Sherbourne C, Wells K. Durham, NC, *Psychological distress/well-being and cognitive functioning measures.* Measuring Functioning and Well-being. , ed. W.J.J. Stewart A. 1992: Duke University Press.
14. Taphoorn, M.J., et al., *An international validation study of the EORTC brain cancer module (EORTC QLQ-BN20) for assessing health-related quality of life and symptoms in brain cancer patients.* Eur J Cancer, 2010. **46**(6): p. 1033-40.
15. Aaronson, N.K., et al., *Translation, validation, and norming of the Dutch language version of the SF-36 Health Survey in community and chronic disease populations.* J Clin Epidemiol, 1998. **51**(11): p. 1055-68.
16. Epskamp, S., D. Borsboom, and E.I. Fried, *Estimating psychological networks and their accuracy: A tutorial paper.* Behav Res Methods, 2018. **50**(1): p. 195-212.
17. Burger, J., et al., *Reporting standards for psychological network analyses in cross-sectional data.* Psychol Methods, 2022.
